# Supplementary material for: Rescue of Aberrant Splicing Caused by a Novel Complex Deep-intronic ABCA4 Allele
Source: Genes (Basel). 2024 Nov 23;15(12):1503. doi: 10.3390/genes15121503 (PMC11675205; doi:10.3390/genes15121503)
Supplement: Supplementary file 1 [file genes-15-01503-s001.zip › Manuscript_ABCA4_int11_Figure_S1-2.pdf]

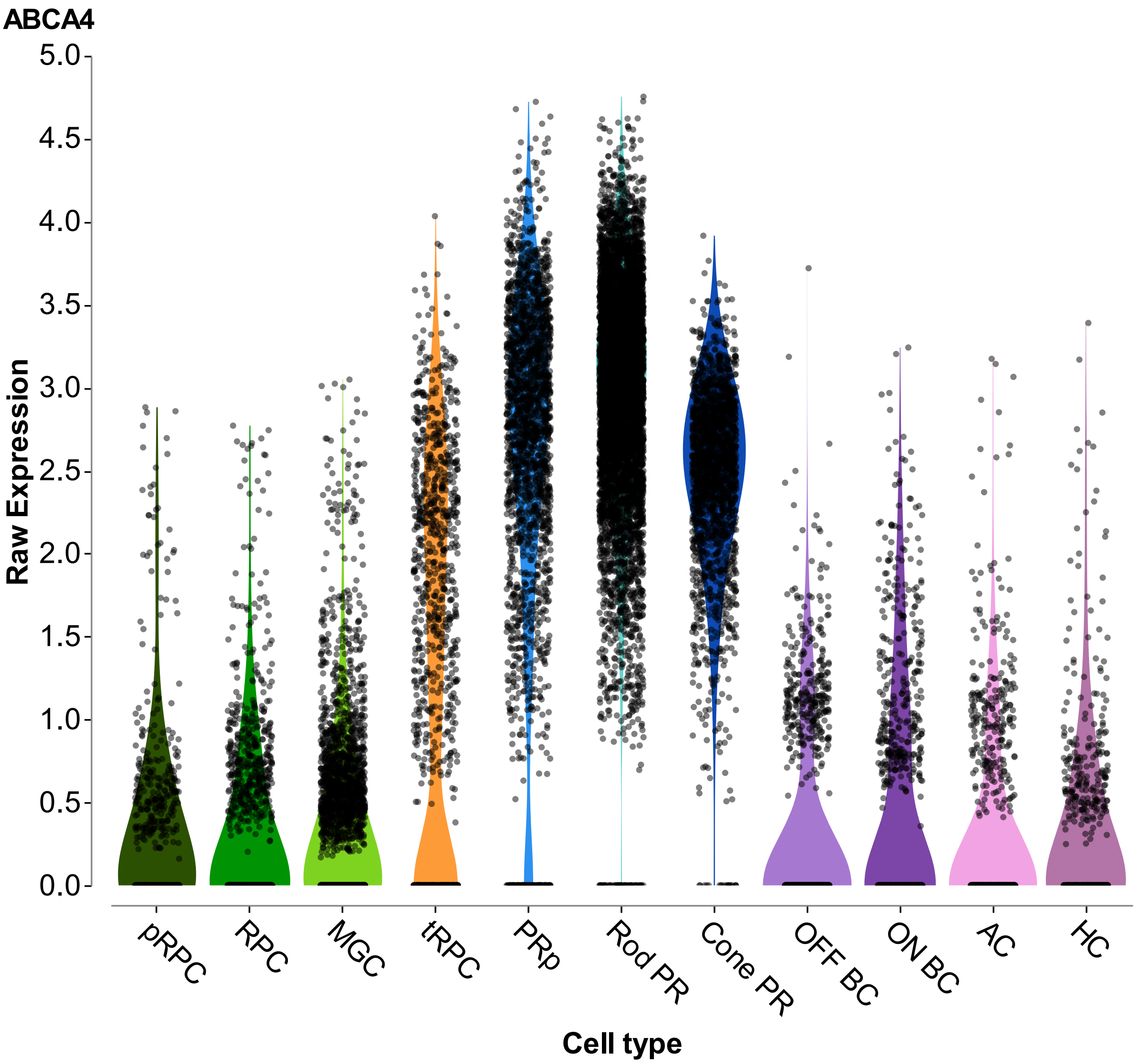

Figure S1. Violin plots generated from single-cell RNA-seq showing *ABCA4* expression in different retinal cell types derived from 23-weeks old "wildtype" retinal organoids generated for a previous study [1]. At this stage of differentiation, a large population of mature rod and cone photoreceptors (Rod PR and Cone PR, respectively) can be detected that robustly expresses *ABCA4*. Each dot overlapping with the violin plot represents a cell. Abbreviations: pRPC, proliferative retinal progenitor cells; RPC, retinal progenitor cells; MGC, Müller glia cells; tRPC, transient retinal progenitor cells; PRp, photoreceptor precursors; Rod PR, rod photoreceptors; Cone PR, cone photoreceptors; OFF BC, OFF-bipolar cells; ON BC, ON-bipolar cells; HC, horizontal cells; AC, amacrine cells.

References

1. Maggi, K.; Atac, D.; Maggi, J.; Feil, S.; Koller, S.; Berger, W. Putative Role of Norrin in Neuroretinal Differentiation Revealed by Bulk and ScRNA Sequencing of Human Retinal Organoids. *bioRxiv* **2024**, 2024.11.15.623746, doi:10.1101/2024.11.15.623746.

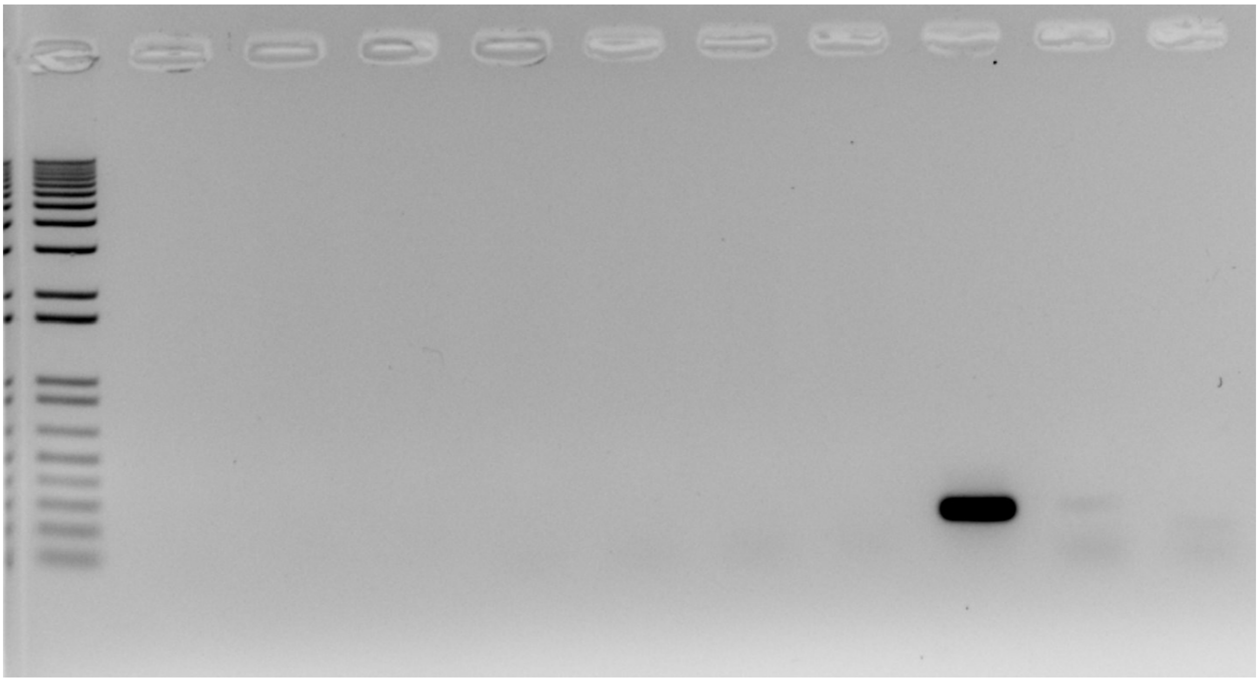

Figure S2. Original full-width gel electrophoresis including the PCRs to detect pseudoexons 11a and 11b from retinal organoids cDNA. A modified version of this image is included in the main body of the article (Figure 8), where the empty lanes of the gel have been removed.
