# Supplementary material for: Rescue of Aberrant Splicing Caused by a Novel Complex Deep-intronic ABCA4 Allele
Source: Genes (Basel). 2024 Nov 23;15(12):1503. doi: 10.3390/genes15121503 (PMC11675205; doi:10.3390/genes15121503)
Supplement: Supplementary file 1 [file genes-15-01503-s001.zip › Table_S1-2.pdf]

Table S1: Splicing prediction scores for cryptic splice sites created or affected by the variants. The effect score has been calculated from the average effect (transformed in percentages) predicted by SSF, MaxEnt, NNSPLICE, GeneSplicer. The distance is meant as basepairs between the variant and the splice site.

Variant (cNomen)	Distance CAS	Distance CDS	Predicted effect CAS	Predicted effect CDS	Effect score CAS (%)	Effect score CDS (%)	SpliceAI AG	SpliceAI DG	Pangolin SG
NM_000350.2:c.[1555-5882C>A;1555-5784C>G]	-41/-139	132/34	Weaker	No effect	-0.20	-0.28	0.02/0.01	0.03/0.05	0.07/0.02
NM_000186.4:c.3494-405A>G	1	83	Stronger	No effect	51.46	0	0	0	0
NM_152778.3:c.199-1334A>G	-304	-5	No effect	Stronger	0	41.83	0.21	0.42	0.3

Abbreviations: cNomen, Human Genome Variation Society (HGVS) cDNA-level nucleotide change nomenclature; CAS, cryptic acceptor splice site; CDS, cryptic donor splice site; AG, acceptor gain; DG, donor gain; SG, splice gain; NA, not applicable.

Table S2: Exonic splicing enhancer and silencer sequences predictions for exon or pseudoexon closest to the variants.

Variant (cNomen)	Distance CAS	Distance CDS	ESS WT	ESS MT	ESE WT	ESE MT	ESS/ESE ratio WT	ESS/ESE ratio MT	EX-skip predicted effect
NM_000350.2:c.[1555-5882C>A;1555-5784C>G]	-41/-139	132/34	50	51	160	178	0.31	0.29	WT higher chance of exon skipping
NM_000186.4:c.3494-405A>G	1	83							Comparable chance of exon skipping
NM_152778.3:c.199-1334A>G	-304	-5							Comparable chance of exon skipping

Abbreviations: cNomen, Human Genome Variation Society (HGVS) cDNA-level nucleotide change nomenclature; CAS, cryptic acceptor splice site; CDS, cryptic donor splice site; WT, reference sequence; MT, mutant (variant) sequence; ESE, exonic splicing enhancer sequence; ESS, exonic splicing silencer sequence; NA, not applicable.
